# Pathophysiology of CD4+ T-Cell Depletion in HIV-1 and HIV-2 Infections

**DOI:** 10.3389/fimmu.2017.00580

**Published:** 2017-05-23

**Authors:** K. K. Vidya Vijayan, Krithika Priyadarshini Karthigeyan, Srikanth P. Tripathi, Luke Elizabeth Hanna

**Affiliations:** ^1^Division of HIV/AIDS, Department of Clinical Research, National Institute for Research in Tuberculosis (ICMR), Chennai, India

**Keywords:** CD4+ T-cell, immune activation, pyroptosis, HIV-1, HIV-2

## Abstract

The hall mark of human immunodeficiency virus (HIV) infection is a gradual loss of CD4+ T-cells and imbalance in CD4+ T-cell homeostasis, with progressive impairment of immunity that leads ultimately to death. HIV infection in humans is caused by two related yet distinct viruses: HIV-1 and HIV-2. HIV-2 is typically less virulent than HIV-1 and permits the host to mount a more effective and sustained T-cell immunity. Although both infections manifest the same clinical spectrum, the much lower rate of CD4+ T-cell decline and slower progression of disease in HIV-2 infected individuals have grabbed the attention of several researchers. Here, we review the most recent findings on the differential rate of decline of CD4+ T-cell in HIV-1 and HIV-2 infections and provide plausible reasons for the observed differences between the two groups.

## Introduction

Acquired immune deficiency syndrome (AIDS) is one of the most devastating infectious diseases affecting humankind, with an estimated 36.7 million people living with human immunodeficiency virus (HIV) infection as per 2015 estimates (1). Although the majority of this infection is caused by HIV-1, a closely related viral strain, HIV-2 that is believed to have spread in parallel with HIV-1 is also an etiological agent of this dreadful infection. The two viruses share striking similarities in genetic and biological properties, such as genome structure and mechanisms for transactivation and CD4+ cell depletion, and yet, HIV-2 exhibits much longer clinical latency periods, significantly lower rates of disease progression and transmission and lower viral load in the asymptomatic phase as compared to HIV-1 infection (2, 3). The distinct differences in pathogenicity provide a unique opportunity to look for protective viral and host immune mechanisms that contribute to viral control.

A significant amount of research has recently been focused on identifying causal factors for the difference in pathogenicity between the two infections in the hope of obtaining clues that could ultimately lead to a sustainable cure in some way. In this context, considerable amount of attention has been paid to understand the hallmark feature of HIV infection, i.e., progressive depletion of CD4 lymphocytes, and its distinct regulation in HIV-infected individuals in whom infection never progresses to AIDS or progresses very slowly. While in the case of HIV-1 infection there is a steady decline in CD4+ T-cell count, in HIV-2 infection, the decline is much slower and viremia levels are lower at any stage of the disease (4–6). In this article, we review the distinct pathological differences between HIV-1 and HIV-2 infections in the perspective of differential rate of CD4+ T-cell decline and provide plausible reasons for the observed differences.

## Depletion of CD4+ T-Cell—A Key Event in HIV Disease Progression

CD4+ T-cells are the central mediators of immune response in humans, crucially coordinating cellular and humoral immune responses against infections. Very early studies on subjects with AIDS documented lymphopenia, low lymphocyte proliferative responses after stimulation with antigens, and an inversion in the ratio of helper T-cells to cytotoxic T-cells (7–9). Further studies in this line confirmed that HIV selectively infects CD4+ T-cells and destroys them for its own benefits (10). Later, it was shown that suppressing HIV replication with antiretroviral therapy (ART) rapidly increased peripheral blood CD4+ T-cell counts and reversed immunodeficiency (11, 12). Now, most researchers agree that HIV majorly infects CD4+ T-cells and leads to progressive loss of the cells from circulation and from the total body stores.

Upon *in vitro* infection with HIV, productive infection of CD4+ T-cells takes place and leads to either cell lysis or giant cell/syncytia formation, in which both infected and uninfected cells fuse, leading to spread of infection (10). Animal models of SIV infection also documented severe depletion of CD4+ T-cells in the gut-associated lymphoid tissue (GALT), which is the major producer of CD4+ T-cells in the body (13). Subsequent studies provided evidence that the same phenomenon of depletion of GALT CD4 reservoirs occurs in human HIV infection as well (13). Quantitative estimates of absolute CD4+ T-cell count and percentage have been shown to correlate strongly with the progression of disease. A normal adult harbors about 22 × 10^11^ CD4+ T-cells (14), whereas in the HIV-infected individual, this number is halved by the time the peripheral blood CD4+ T-cell count falls to 200 cells/microliter of blood (14, 15). In more advanced disease, destruction of parenchymal lymphoid spaces is so extensive that enumeration of the total body CD4+ T-cell count cannot even be attempted. Since HIV induces both quantitative and qualitative defects in the CD4+ T-cell compartment, numbers of circulating CD4+ T-cells in HIV+ subjects have been the most widely used tool for predicting the onset of overt immunodeficiency and the best surrogate marker for monitoring severity of the disease (16).

## Contributors to CD4+ T-Cell Depletion

CD4+ T-cells are known to be the central facilitators for both cellular and humoral immune responses against exogenous antigens and are kept constant in the human body by homeostatic mechanisms (17, 18).HIV binds to the CD4 molecule on the surface of helper T-cells and replicates within them. This results in destruction of CD4+ T-cells and leads to a steady decline in this population of T-cells. The definition of progressive and slow loss of CD4+ T-cells is not clear. In order to understand the correlation between CD4+ T-cell depletion and immunopathogenesis, and its relationship with disease progression, a number of dynamic models have been put forward. Two of the most acknowledged mechanisms are discussed in detail in this review. These include direct virus attack leading to cytolytic effect and chronic immune activation resulting in apoptosis.

### Direct Attack by HIV

Several studies carried out in the late 1980s and early 1990s provided support for the hypothesis of “accelerated destruction” of CD4+ T-cells by viral attack. This hypothesis received indirect experimental validation from Ho and colleagues (19, 20), who proposed “the tap and drain hypothesis” for the slow depletion of CD4 cells. According to this theory, there is a homeostatic response by which the loss of CD4+ T-cells due to HIV infection (the drain) is comfortably counteracted by production of T-cells (a wide open tap); however, this balance is ultimately disrupted once the production of T-cells in response to homeostasis is exhausted. This has been substantiated by quantitative image analysis of decreased numbers of CD4+ T-cells and increased levels of cellular proliferation and apoptosis in HIV-infected individuals (21, 22).

Given the fact that HIV infection accelerates both the production and the destruction of CD4+ T-cells, in the early stages of the infection, there is constant replacement of dying CD4+ T-cells with native CD4+ T-cells originating from the thymus (21). It is reported that during the course of HIV infection, about 1 billion of HIV particles are produced per day, resulting in increasing numbers of infected CD4+ T-cells (21, 23). Subsequently, infection spreads to the memory cells in the thymus and the virus starts to replicate there. Each time a memory CD4+ T-cell is infected by HIV, it is destined to undergo the process of elimination, thus contributing to the progressive decline in CD4+ T-cell numbers (22). Analysis of T-cell turnover in humans with HIV infection suggests that the fraction of dividing CD4+ T-cells in untreated HIV disease can be elevated two- to threefold (24, 25), with most proliferation concentrated in the CD45RO+ memory/effector population of CD4+ T-cells (26). While direct viral killing/cytolysis of CD4 T-cells partly clarifies the cause of depletion of CD4+ T-cells, the loss of uninfected CD4 cells and naïve CD8 cells during the asymptomatic phase of HIV infection cannot be explained by this hypothesis. Taken together, these observations suggest that AIDS pathogenesis cannot solely be explained by the direct viral killing hypothesis.

### Chronic Immune Activation

Another dynamic model that emerged to explain the pathogenesis of AIDS and accompanying CD4 depletion is the “hyper immune activation hypothesis,” which suggests that there is a high rate of cell division among the CD4+ and CD8+ T-cell, NK cell, and B cell populations during the course of HIV infection and an associated upregulation of activation markers (27). Several studies have demonstrated that the level of immune activation in HIV-infected subjects is a better predictor of disease progression than the levels of virus replication (28, 29). Hyperimmune activation induces increased cell division in memory T-cells and increases their capacity for self-renewal, thereby leading to increased cell counts. To explain “depletion by activation,” Yates et al. proposed that the activated CD4+ T-cells have a very short life span and are lost rather quickly due to activation-induced cell death or apoptosis(30). Labeling studies have shown evidence of increased turnover of naïve T-cells in HIV infection. This is also supported by the dilution of excision circles of the T-cell receptor (31). Thus, chronic immune activation better explains immune deficiency among HIV-infected individuals, and the level of activation predicts disease progression in HIV-infected subjects better than viral replication-driven pathogenesis (32–34).

Investigation on HIV infection has shown that immune system remains in a hyperactive state characterized by high T-cell turnover, non-specific T-cell activation and proliferation, polyclonal activation of B cells, and elevated proinflammatory cytokines (35). Thus, HIV makes its own target and increases replication through immune activation. HIV infection activates the immune system through the viral gene products Nef, Tat, Vpr, and Vpu (30, 36) and also through production of inflammatory cytokines (37). For instance, Nef and Vpr are involved in stimulating monocytes and macrophage cells (37). Likewise, plasmacytoid dendritic cells (DCs) are induced by HIV RNA; they interact with the pattern recognition receptors such as toll-like receptor (TLR)-7 and TLR-9 and induce the production of interferon (IFN)-α (38). In addition, the presence of HIV DNA in the cytoplasm itself leads to the activation of caspase-1 and to the release of proinflammatory cytokines including interleukin (IL)-1β (38). Thus, even abortive HIV-1 infection, if it results in the presence of viral DNA in the cytoplasm of target cells, could induce immune activation.

### Immune Activation and Inflammation

In general, HIV-associated chronic immune activation is characterized by high levels of circulating proinflammatory cytokines and chemokines, including type I IFNs, IL-6, TGFβ, IL-8, IL-1α, IL-1β, MIP-1α, MIP-1β, and RANTES (39, 40). Plasma proteins such as neopterin, β2-microglobulin (β2M), TNF-α, soluble TNFRII, soluble IL-2R, and IFN-γ are documented to be increased even in the early stages of infection (34, 40). Many other proteins, including anti-inflammatory cytokines IL-10 and TGF-β1 and IFN-inducible protein-10 (IP-10), have also been shown to be increased in the plasma during acute infection and are reported to be predictive of rapid disease progression (41). Thus, massive increase of cytokine release, called cytokine storm, characterizes acute and chronic HIV infection and contributes to predict the immune activation and CD4+ T-cell depletion. Persistent immune activation also prevents the establishment of IL-2-producing memory CD4+ and CD8+ T-cells (42), and this has deleterious effects on HIV-specific CD4+ T-cell immunity (43).

During early infection, the virus primarily disturbs the mucosal immune function. Loss of integrity of the gastrointestinal mucosa and microbial translocation contribute to induction of local inflammation and HIV-associated chronic immune activation, which in turn are associated with disease progression and CD4+ T-cell depletion (44). Several studies have shown that poorly controlled translocation of immunostimulatory microbial products occurs in HIV-infected individuals. Through the stimulation of several TLRs, these microbial products activate various immune cell types and induce production of proinflammatory cytokines, such as TNF-α, IL-6, IL-1β, and type I IFNs (44–46). These responses significantly contribute to the aberrant immune activation in chronic HIV infection. The breach of the gut system has been shown to correlate well with the level of immune activation and depletion of CD4+ Th17 cells, a cell type that is normally abundant in the mucosa and is known to be involved in immunity to commensal bacteria (47). The selective loss of Th17 CD4+ T-cells from the gut possibly due to selective infection has therefore been held responsible for the long-term loss of the intestinal integrity and thereby for chronic immune activation in pathogenic HIV infection (47–49). In short, HIV infection is characterized by massive production of proinflammatory cytokines (48, 49), which in turn leads to clonal deletion (49) and gradual loss of peripheral CD4+ T-cells over time (50).

### Pyroptosis and Apoptosis

It has been known for some time now that apoptosis is a major factor contributing to T-cell depletion, mediated by *caspase-3*, in T-cells that are permissive to infection by HIV. However, there are subsets of T-cells (abortive T-cells) that are non-permissive and do not support HIV replication. In this subset of cells, cell death occurs through a process called pyroptosis, driven by *caspase-1* (51). Pyroptosis is a highly inflammatory form of programmed cell death in which the dying cell releases all its cytoplasmic contents, including inflammatory cytokines; these cytokines in turn trigger pyroptosis in other T-cells as part of a vicious cycle of abortive T-cell depletion (52). Recent studies by Doitsh et al. showed that depletion of T-cells and subsequent progression of disease occur not only through apoptosis but also through pyroptosis (51). In a subsequent study, the same group of investigators showed that only 5% of CD4+ T-cell depletion occurs through apoptosis, while the remaining 95% of quiescent lymphoid CD4+ T-cells die by caspase-1-mediated pyroptosis triggered by abortive viral infection (51, 52). Pyroptosis thus links the two signature events in HIV infection—CD4+ T-cell depletion and chronic inflammation—and creates a pathogenic vicious cycle in which dying CD4+ T-cells release inflammatory signals that stimulate more cells to die. Thus, it establishes a state of chronic inflammation that eventually fuels disease progression.

### Regulatory T-Cells (Treg)

CD4+ Treg gained prominence recently. These cells play a vital role in T-cell homeostasis and limiting of immunopathology through the suppression of specific T-cell responses such as activation, proliferation, and effector function (53). A study undertaken by Foxall et al. in 2011 demonstrated that CD4 depletion is associated with relative expansion of Treg cells, irrespective of the presence or absence of circulating virus, leading to better preservation of circulating naive and memory Treg cells as compared to other CD4+ T-cell subsets in HIV/AIDS (54). Increased frequency of CD4+ T-cells expressing CD25 has been observed in HIV-2-infected individuals, independent of the degree of CD4 depletion and levels of immune activation. CD4+CD25+ T-cells, a subset that expresses the highest level of the lineage regulatory marker FoxP3, are proposed to play a significant role in models of experimental chronic infection by contributing to suppression of T-cell responses (55). On the other hand, CD4+ T-cells expressing an intermediate intensity of CD25 are characterized by an increased ability to produce IL-2 and lack other regulatory markers. The expansion of this population of cells is not seen in HIV-1 infection. The presence of higher levels of these cells in HIV-2-infected individuals may point to an improved ability to replenish their CD4 memory pool and to the lower virulence of HIV-2 infection as compared to that of HIV-1. Furthermore, the Treg cell population can be subdivided into CD45RA+ (naïve-resting cells) and CD45RA− (memory-activated cells). The majority of Tregs in the HIV-2-infected individuals have been observed to lack CD45RA expression, thus implying that most of the Tregs in these individuals belong to the memory-activated phenotype. These findings further support a close link between CD4 depletion and immune activation. Thus, it is plausible that Tregs also have a role in the slower rate of disease progression associated with HIV-2 infection (54, 56).

## Distinguishing Features of CD4+ T-Cell Depletion in HIV-1 and HIV-2 Infections

One of the best ways of elucidating the intriguing nature of immunopathogenesis of HIV infection is studying naturally occurring HIV infection with different clinical outcomes. HIV-2 infection provides an ideal situation for this investigation as it has a lower degree of pathogenicity as compared to HIV-1. Although HIV-2 also eventually causes immunodeficiency syndrome indistinguishable from HIV-1-induced AIDS (57, 58), many HIV-2-infected individuals do not develop immunodeficiency during their lifetime and retain stable CD4+ T lymphocyte counts and low levels of viremia for many years (4–6). This striking difference has prompted the search for the reason for variations in T-cell homeostasis and imbalances in cytokine production and identification of factors that contribute to an effective immune response that delays progression of disease during infection.

One of the major aspect of CD4+ T-cell depletion and its associated immunopathology that distinguishes between HIV-1 and HIV-2 infections is immune activation (59), which is a strong predictor of disease progression HIV infection (60). One of the key drivers for immune activation during chronic HIV-1 and HIV-2 infection is the breach of the gastrointestinal tract, resulting in translocation of bacterial lipopolysaccharide (LPS) into the blood (28, 34). LPS is a known activator of innate immune cells *via* TLR-4, and LPS concentrations in the circulation of HIV-infected individuals correlated strongly with T-cell activation levels (61, 62). A study by Nowroozalizadeh et al. showed an inverse correlation between plasma LPS levels and expression of proinflammatory cytokines IL-12 and IFN-γ following TLR stimulation in HIV-2-infected, HAART-naïve individuals, whereas in HIV-2-infected individuals with AIDS, there was a positive correlation between LPS levels, CD4+ T-cell lymphopenia, and HIV RNA load similar to HIV-1 infection (63).

The viral proteins Nef, Env, and Tat also play a part in immune activation. In HIV-2 infection, intracellular Nef promotes the downregulation of the T-cell receptor complex in CD4+ T-cells, whereas in HIV-1 infection, Nef seems to have lost its ability to downmodulate the expression of CD3–TCR complex on the surface of infected T-cells (64). As a consequence, HIV-1 Nef may directly contribute to immune activation by rendering infected CD4+ T-cells highly sensitive to restimulation through the T-cell receptor. In contrast, the low level of immune activation in HIV-2-infected individuals with low virus replication may prevent the shift in T-cell function and phenotype in chronic infection. Studies have also shown that low levels of circulating virus lead to low levels of activation of CD4 and CD8 cells; gag mRNA level has been found to correlate with CD4+ T-cell activation and tat mRNA with CD8+ T-cell activation (65, 66). Studies have also shown that tat mRNA transcripts accumulate and outnumber gag mRNA transcripts in recently infected cells with HIV-1 (67), whereas HIV-2 infected cells had reduced levels of tat mRNA transcripts, indicating a decreased rate of *de novo* cell infection in HIV-2 disease (66). This lower level of immune activation in HIV-2 cohorts as compared to HIV-1-infected groups (67) may explain the relative sparing of T lymphocytes from cell death in HIV-2 infection, which is consistent with the “less activation/better outcome” paradigm.

The function of the thymus is well preserved in HIV-2-infected individuals, allowing CD4+ T-cells to retain better proliferative capacity, remain less differentiated, and elicit more polyfunctional responses, than in HIV-1 infected individuals (68). HIV-2-infected individuals are also highly efficient in replacing infected CD4+ T-cells (69). Earlier studies have revealed the central role of IL-2 and IFNγ as survival and proliferative factors (70, 71) in HIV-1 infection; as disease progresses, the frequency of IL-2-producing CD4+ T-cells is found to decline (42), which in turn relates to the reduced renewal capacity and increased susceptibility of these populations of cells to apoptosis (70). However, in HIV-2 infection, the proportion of CD4+ T-cells expressing IL-2 is well preserved (42). HIV-2-infected individuals also possess a higher frequency of CD4+ T-cells capable of expressing both IFNγ and IL-2 in response to Gag-specific peptides than HIV-1 infected individuals (72, 73). Further immunological studies revealed the presence of Gag-specific CD4+ T-cells lacking CD57 expression in HIV-2 infection (74, 75), indicating better proliferative capacity resulting in higher number of cells in HIV-2 infected individuals (75). In lieu with these observations, normal CD4 counts in HIV-2-infected individuals were three times greater than in HIV-1-infected individuals with comparable levels of CD4+ T-cells (74). These findings provide conclusive evidence for the lower lymphocyte susceptibility to apoptosis and slower rate of CD4+ T-cell decline in HIV-2-infected individuals as compared to those with HIV-1 infection.

## Concluding Remarks

With decades of experimental research and volumes of observational data, the complete mechanism of CD4+ T depletion in HIV infection still remains to be explained. HIV-2, a natural model of attenuated HIV infection, provides an appropriate system for exploring paradigms for pathogenesis and helps in understanding retroviral pathology. This review highlights the unique aspects of CD4+ T-cell depletion in HIV-1 and HIV-2 infections (Table 1). Collectively, the available data suggests that HIV-2 is associated with more efficient immunologic responses and lower replication efficiency in primary cells including resting CD4+ T-cells and activated CD4+ T-cells which ultimately leads to better virus control and slower disease progression.

**Table 1:** **Comparison of HIV-1 and HIV-2 infection**.

	Human immunodeficiency virus (HIV)-1	HIV-2
**Epidemiological and clinical significance between HIV-1 and HIV-2 infection**		

Geographical distribution	Global	Confined to West Africa with limited spread; also reported in former Portuguese colonies, such as Angola, Mozambique, and Brazil, and in parts of India

Heterosexual transmission	Sexual mode of transmission is higher	Fivefold lower rate than HIV-1

Vertical transmission	Mother to child transmission is higher	20- to 30-fold lower rate than HIV-1

Duration of asymptomatic stage	Time to develop acquired immune deficiency syndrome (AIDS) varies, ranging from a few months to many years, with an estimated median time of 9.8 years	Longer duration, ranging from 10–25 years

Clinical illness	If untreated, around half of people infected with HIV-1 will develop AIDS within 10 years	86–95% of people infected with HIV-2 are long-term non-progressors

Proviral DNA load	Similar	Similar

Plasma RNA load	Higher	Significantly lower than HIV-1

Viral replication kinetics	Higher replication and 100-fold more fit	Transient replication and less fit

Infectivity and transmission fitness	Similar and 100-fold more fit	Similar and less fit

Co-receptor usage	Uses CXCR4 and CCR5	Uses a range of co-receptors, including CCR1, CCR2, CCR3, CXCR6, BOB, CCR5, and CXCR4

**CD4+ T-cell responses between HIV-1 and HIV-2 infection**		

CD4+ T-cell count	Lower compared to HIV-2 with undetectable viral load but similar to HIV-2 with higher viral load	Higher in HIV-2 with undetectable viral load and similar to HIV-1 with higher viral load

CD4+ T-cell response	Lesser proliferative capacity and polyfunctionality, and increased differentiation	Better proliferative capacity, more polyfunctionality, and lesser differentiation

Thymic function	HIV-1 can replicate efficiently in thymus tissue. No correlation with the rate of CD4+ T-cell loss	HIV-2 is able to infect the human thymus, but this is associated with limited viral replication. Correlates with lower rates of CD4+ T-cell

Production of cytokines	Interleukin (IL)-2- and IL-4-producing cells decline as disease progresses	IL-2- and IL-4-producing cells better preserved. Expressions of both IL-2- and IFN-γ-producing cells are more

CD57−CD4+ T-cell expression	Less frequently seen	More CD57− cells are seen

CD4+ T-cell activation level	Positive correlation between lipopolysaccharide (LPS) level and proinflammatory cytokines IL-12 and IFN-γ	Negative correlation between LPS level and proinflammatory cytokines in HIV-2 individuals with undetectable VL

Susceptibility to the SAMHD1	Myeloid cells are refractory to viral infection	Presence of Vpx permits viral infection of myeloid cells through degradation of SAMHD1

Immune activation and T-cell apoptosis	Higher immune activation and more apoptosis	Less immune activation and less T-cell apoptosis

Nef	Does not downmodulate the TCR–CD3 complex	Downregulates the TCR–CD3 complex

Recent studies have identified several other factors that contribute to the difference in pathogenesis between HIV-1 and HIV-2 infections. One of these includes cellular sensors that are involved in the recognition of HIV and induction of innate and adaptive immune responses (76). Certain key differences between innate sensing mechanisms in HIV-1 and HIV-2 infections have been identified. One of the major cell types involved in differential innate immune sensing between HIV-1 and HIV-2 is the DCs (77). DCs do not normally get activated and are not efficiently infected by HIV-1 (78, 79), since SAMHD1, which is an intracellular exonuclease, restricts HIV replication and transcription in dendritic and myeloid cells by hydrolyzing deoxynucleoside triphosphates during reverse transcription of viral RNA (79). In contrast, DCs are naturally activated and infected by HIV-2 (80); the Vpx protein that is produced by HIV-2 and not by HIV-1 is responsible for overcoming the SAMHD1 restriction in DCs (77, 80). Intrinsic pattern recognition receptors within the DCs sense HIV-2. The signals from the PRRs direct the antigen-presenting DCs to mature and generate effective acquired immune responses against HIV-2. This view is supported by the finding that polyfunctional, virus-specific, T-cell responses are more commonly seen in HIV-2 than in HIV-1 infection (80). SAMHD1 degradation thus could have both positive and negative effects on the efficiency of HIV-2 replication *in vivo*. On the one hand, it allows efficient infection of macrophages and DCs with HIV-2, thus expanding the number of viral target cells, and on the other hand, SAMHD1 degradation by Vpx may contribute to the effective control of viral replication in HIV-2-infected individuals by inducing potent immune responses through viral immune sensing by infected DCs (81).

The ability of HIV to seed latent reservoirs in the body very early during infection is one of the major barriers for eradication of the virus. The stable latent reservoirs in the body comprise long-lived, transcriptionally inactive, and immunologically inert cells lodged in anatomical sites with poor drug penetration. The major constituents of the viral reservoir are the latently infected resting memory CD4+ T-cells and macrophages (82, 83). HIV latency can be of two types: pre-integration and post-integration. Although not much is known about the contribution of pre-integration latency to HIV-2 infection, post-integration HIV-2 latency, through post-transcriptional control of viral replication, has been described after *in vitro* infection of specific subsets of target cells (84). Subtle differences in the transcriptional control elements present in the HIV-2 LTR are thought to contribute to the differences in transcriptional activity and, in a way, to the differences in the pathogenesis between the two viruses. More studies in this line will help to further clarify the mechanisms that contribute to better control of HIV-2 infection and pave way for the design of effective strategies to prevent disease progression in HIV-infected individuals.

## Author Contributions

KV drafted the manuscript. KK helped with literature collections. ST and LH reviewed the manuscript.

## Conflict of Interest Statement

The authors declare that the research was conducted in the absence of any commercial or financial relationships that could be construed as a potential conflict of interest.
